# Occult endometrial pathologies diagnosed after sacrocolpopexy with concomitant supracervical hysterectomy for pelvic organ prolapse

**DOI:** 10.3389/fsurg.2026.1704824

**Published:** 2026-02-02

**Authors:** Sarah Marcelle, Michelle Nisolle, Katty Delbecque, Frederic Goffin, Laurent De Landsheere

**Affiliations:** 1 Department of Gynecology-Obstetrics, University of Liège, CHU Liège, Liège, Belgium; 2 Department of Anatomo-Pathology, University of Liège, CHU Liège, Liège, Belgium

**Keywords:** morcellation, occult malignancy, pelvic organ prolapse, sacrocolpopexy, supracervical hysterectomy

## Abstract

**Introduction:**

Sacrocolpopexy with concomitant hysterectomy is a common procedure for the treatment of pelvic organ prolapse. However, occult endometrial pathologies, including malignancies, may be present in some patients, which should be considered when planning the management. The objective of this study was to determine the incidence of occult endometrial pathologies, including malignancies, in patients undergoing sacrocolpopexy with concomitant supracervical hysterectomy, and to identify associated risk factors.

**Materials and methods:**

This was a retrospective, single-center study including all patients who underwent sacrocolpopexy with supracervical hysterectomy between November 2010 and December 2023. A total of 226 procedures were analyzed.

**Results:**

Among the 226 patients, 15 cases of occult endometrial pathology were identified, representing an incidence of 6.6%. These included 14 cases of endometrial hyperplasia with atypia (6.2%) and 1 case of endometrial carcinoma (0.4%). No oncological recurrence was observed after a mean follow-up period of 26 months.

**Conclusions:**

The incidence of occult endometrial pathologies in this cohort was 6.6%. Particular attention should be given to postmenopausal patients and those with risk factors for endometrial malignancy. The use of a containment bag is recommended during specimen extraction to prevent the potential dissemination of occult malignancies. This study highlights the importance of avoiding unprotected morcellation of surgical specimens, particularly in high-risk populations.

## 1 Introduction

Pelvic organ prolapse (POP) is a common gynecological condition, which its incidence increases with age and affecting approximately 50% of the female population. It can impair their quality of life (QOL) and be associated with urinary or anorectal symptoms (1, 2). Therapeutic options for POP include conservative treatments, such as pessaries and pelvic floor rehabilitation, as well as surgical interventions, which can be performed through vaginal, laparoscopic, or robotic approaches. Recently, there has been a shift towards sacrocolpopexy, which is considered the gold standard surgery for treating apical prolapse (3). This procedure can also be combined with a hysterectomy in cases of uterine prolapse, uterine pathologies, or when patients present with symptoms such as pain, metrorrhagia, or menorrhagia (2, 4–6).

The benefits of supracervical hysterectomy with cervical preservation, as compared to total hysterectomy during sacrocolpopexy, include reduced blood loss, decreased exposure to prosthetic materials, and lower incidence of urinary tract injuries (2, 6). When performing supracervical hysterectomy, the method of specimen extraction is crucial. Morcellation of the uterus reduce incision size and surgical morbidity; however, it carries a risk of intra-abdominal dissemination of an occult malignancy if a containment bag is not used (7).

Despite a thorough preoperative assessment, preinvasive or invasive lesion may incidentally be discovered following the pathological analysis of surgical specimens. Limited data is available in the literature on the incidence of occult endometrial lesions, with reported rates ranging from 0.2% to 3.2% (2, 8–12).

The primary objective of this study is to determine the incidence of occult endometrial pathology in patients undergoing sacrocolpopexy with concomitant supracervical hysterectomy. The secondary objective is to identify potential risk factors for patients with occult malignancy. Additionally, we will report the outcomes of patients in whom a preinvasive or invasive lesion was discovered, along with the appropriate management for such diagnoses.

## 2 Methods

This monocentric descriptive case series includes all consecutive patients who underwent a supracervical hysterectomy during sacrocolpopexy between November 2010 and December 2023 at the Department of Gynecology and Obstetrics of CHU de Liège, Site de la Citadelle. All surgeries were performed by the same gynecologist surgeon (LdL). The study was approved by the internal ethics committee, under reference number 2177.

The demographic data for each patient, including age, body mass index (BMI), personal history of breast neoplasia treated with hormone therapy (Tamoxifen), presence of type 2 diabetes or hypertension, menopausal status and presence of menorrhagia or metrorrhagia were collected and analyzed.

The preoperative assessment included a cervical cytology (Pap smear) and a pelvic ultrasound for all patients.

Patient with abnormal Pap test were referred to the colposcopy clinic and were treated for their cervical lesion prior to POP surgery.

Patients with abnormal uterine bleeding and/or abnormal endometrial thickness underwent endometrial biopsy, with hysteroscopy performed when indicated. *Specifically,* a threshold of 5 mm was considered pathological in women with postmenopausal bleeding, while a threshold of 11 mm was applied in asymptomatic postmenopausal women. The operative data included the type of primary surgical procedure, any associated procedures (such as oophorectomy, placement of a suburethral sling), the method of specimen extraction (contained in-bag intact extraction, intracorporeal power morcellation or contained in-bag morcellation), the surgical approach (laparoscopic or robotic), as well as the weight and analysis of the surgical specimens. All analyses were performed by the same pathologist (KD), an expert in gynecological pathology and the pathologies were classified according to the WHO 2020 classification (13).

Patients with an incidental diagnosis of preinvasive or invasive endometrial pathology were discussed in a multidisciplinary team meeting, coordinated by a gynecologic oncologist (FG), and including a medical oncologist, a radiation oncologist, a pathologist, a radiologist, and a nurse coordinator.

Statistical analyses were performed as follows. Descriptive statistics were provided for each baseline variable. To compare the variances between the two groups, we used the Fisher-Snedecor test. A *p*-value <0.05 was considered statistically significant.

## 3 Results

A total of 226 patients were treated by sacrocolpopexy combined supracervical hysterectomy between November 2010 and December 2023. All patients were included in the study.

The demographic data are summarized in Table 1. Patients with occult endometrial atypia or neoplasia were, on average, older (66.3 years) compared to those without lesions (59.1 years) (*p* = 0.03). All patients with lesions were postmenopausal, compared to 69.7% of patients without lesions (*p* = 0.007). Their body mass index was similar between the two groups. A higher proportion of patients with lesions had a history of tamoxifen hormone therapy (13.3% vs. 1.9%) (*p* = 0.05). Type 2 diabetes was present at similar rates in both groups (18.5% vs. 20%) (*p* = 1). However, hypertension was significantly more frequent in patients with lesions (53.3% vs. 23.7%) (*p* = 0.017). Lastly, symptoms of menorrhagia or metrorrhagia were more frequently reported in patients with lesions compared to those without (13.3% vs. 7.1%), although this difference did not reach statistical significance (*p* = 0.3).

**Table 1:** Demographic data of patients with and without occult endometrial atypia/neoplasia.

Characteristics	Patients without endometrial atypia/neoplasia (*n* = 211)	Patients with occult endometrial atypia/neoplasia (*n* = 15)	*P*-value
Mean age (years), range	59.1 (33–84)	66.3 (55–81)	0.03
Mean BMI (kg/m^2^), range	25.7 (15–38)	26.3 (21–37)	0.52
History of hormone therapy (tamoxifen)	4 (1.9%)	2 (13.3%)	0.05
Type 2 Diabetes	39 (18.5%)	3 (20.0%)	1
Hypertension	50 (23.7%)	8 (53.3%)	0.017
Menorrhagia/metrorrhagia	15 (7.1%)	2 (13.3%)	0.3
Menopause	147 (69.7%)	15 (100%)	0.007

In this study, preoperative Pap smears were performed and documented for all 226 patients (100%). Cervical abnormalities were investigated by colposcopy with or without directed biopsies and, when indicated, managed before sacrocolpopexy.

All 226 patients (100%) underwent preoperative pelvic ultrasound. Findings were normal in 166 (73.5%). Among the remaining 60 (26.5%), benign conditions—fibroids, adenomyosis, polyps, or ovarian cysts—were identified. Six patients (2.7%) showed endometrial thickening; in line with our protocol thresholds (endometrial thickness ≥5 mm in symptomatic postmenopausal women and ≥11 mm in asymptomatic cases), they underwent further evaluation with hysteroscopy and endometrial biopsy, which excluded preinvasive or invasive disease.

Seventeen patients (7.5%) reported menorrhagia or metrorrhagia; among them, hysteroscopy was performed in 3 cases (17.6%) and endometrial biopsy in 9 (52.9%), all of which were benign. Overall, hysteroscopy was carried out in 13 patients without endometrial atypia/neoplasia (6.2%) and in none of the patients with occult atypia/neoplasia, while endometrial biopsy was performed in 36 patients without atypia/neoplasia (17.1%) and in 3 patients with occult atypia/neoplasia (20%). *None of these additional investigations revealed malignant disease.*

Details of the interventions are summarized in Table 2, and the various pathological diagnoses are listed in Table 3. Among the 226 hysterectomy specimens with salpingectomy, 204 (90.3%) had one or more benign pathologies, 14 (6.2%) had endometrial hyperplasia with atypia, and one (0.4%) had a grade 1 endometrial carcinoma according to the International Federation of Gynecology and Obstetrics (FIGO) classification. All surgical margins were clear.

**Table 2:** Operative data of patients with and without occult endometrial atypia/neoplasia.

Operative data	Patients without endometrial atypia/neoplasia (*n* = 211)	Patients with occult endometrial ypia/neoplasia (*n* = 15)
Laparoscopy	159 (75.4%)	10 (66.7%)
Robot-assisted laparoscopy	52 (24.6%)	5 (33.3%)
Power morcellation	82 (38.9%)	2 (13.3%)
Contained morcellation	50 (23.7%)	1 (6.7%)
No morcellation	79 (37.4%)	12 (80%)
Concomitant oophorectomy	159 (75.4%)	13 (86.7%)
Concomitant sling surgery	59 (28%)	5 (33.3%)
Mean follow-up (months)	19	26

**Table 3:** Histological analysis of specimens obtained during sacrocolpopexy with hysterectomy.

Histology	Number of specimens (*n* = 226)
Uterus	
Mean weight (g)	81,8
Leiomyoma	97 (42.9%)
Adenomyosis	131 (58.0%)
Non-benign myometrial diseases (STUMP, leiomyosarcoma)	0 (0%)
Endometrium	
Simple hyperplasia	50 (22.1%)
Polyp	39 (17.3%)
Hyperplasia with atypia	14 (6.2%)
Endometrial carcinoma (FIGO stage 1)	1 (0.4%)
Ovaries	
Benign tumor	7 (3.1%)
Malignant/ borderline tumor	0 (0%)
Fallopian tubes	
Benign pathology	12 (5.3%)

All 15 patients with endometrial lesions had undergone a preoperative pelvic ultrasound and Pap smear, neither of which demonstrated any pathology. Two patients, both with very focal atypia, underwent morcellation without the use of a containment bag. Following these two cases, from January 2017, all morcellation were performed within a containment bag. The patient with grade 1 endometrial carcinoma according to FIGO classification had her specimen extracted in a containing without morcellation.

Among the 14 patients diagnosed with endometrial hyperplasia with atypia, the two first patients (14.3%) who undergone power uncontained power morcellation of the specimen, received progestin therapy. Gynecological follow-up, including clinical and ultrasound monitoring, was recommended by the multidisciplinary team without immediate reintervention. For the patient with an occult endometrial invasive adenocarcinoma FIGO stage I, the reassuring surgical findings combined with a negative MRI staging assessment led to the decision of close follow-up with regular clinical and radiological surveillance, rather than trachelectomy, which was considered too invasive given the patient's advanced age (80 years). A CT scan performed two years after surgery showed no evidence of recurrence.

Additionally, patients with preinvasive or invasive endometrial lesions were followed for a mean duration of 26 months, with no evidence of oncological recurrence.

## 4 Discussion

In the literature, the incidence of incidental discovery of preinvasive or invasive endometrial lesion during hysterectomy for POP ranges from 0.2% to 3.2% (2, 8–12). The most important finding of our study is the higher overall rate of endometrial lesions compared to these reported in the literature. This likely reflects centralized histopathologic assessment: all specimens were reviewed by a single experienced gynecologic pathologist using uniform criteria and a rigorous, systematic evaluation, which may have increased detection of subtle lesions. Notably, most of these lesions are preinvasive, while the rate of occult invasive endometrial adenocarcinoma is lower than what has been previously reported (2, 8–10, 12).

The follow-up of these 15 patients, with a mean duration of 26 months, was reassuring, as none required reintervention and no neoplastic recurrence was observed. These findings contrast with previous studies, where reintervention was often necessary, mostly due to the absence of specimen morcellation. Following the first two incidental findings of preinvasive endometrial lesions, our practice was modified: from January onward, no specimen was subjected to uncontained power morcellation.

Despite the 2014 FDA safety communication highlighting the risk of tumor dissemination associated with uterine morcellation, the systematic use of containment bags during benign gynecologic surgery has not been widely adopted. According to a recent study by Kim R. et al., the use of a containment bag during hysterectomy for benign disease remains limited and shows significant regional variations: only 18% of surgeons in the United States reported using it, compared with 57% internationally. Among those performing morcellation, the use of a containment bag was reported in approximately two-thirds of cases in the United States (66%) vs. only half of the cases internationally (51%) (14, 15).

In postmenopausal women, transvaginal ultrasound assessment of the endometrium is a key tool for detecting endometrial pathology. According to the recommendations of the International Endometrial Tumor Analysis (IETA) group and data from the literature, a threshold of 5 mm is generally used in the case of postmenopausal bleeding: below this threshold, the risk of endometrial cancer is <1%, while it rises to approximately 7% above it (16).

In asymptomatic women, an endometrial thickness ≤ 11 mm is associated with a very low risk (< 0.1%), whereas an endometrial thickness > 11 mm carries an estimated risk of about 6.7% (17–19).

In our study, we relied on these criteria for preoperative selection. Nevertheless, we still observed incidental findings of endometrial lesions, highlighting the importance of rigorous preoperative evaluation and supporting the systematic use of protective measures such as specimen morcellation within a containment bag (endobag), even when preoperative investigations appear reassuring. Several demographic factors, including advanced age, hypertension, postmenopausal status and the use of hormone therapy for breast neoplasia, are associated with an increased risk of occult pathologies.

Our study has several strengths, including its long duration and the extended follow-up period. All patients who underwent sacrocolpopexy with hysterectomy during this time were included, and all surgical specimens were analyzed by the same expert pathologist, ensuring consistency in surgical technique and diagnostic interpretation, thereby minimizing potential variability and bias. However, this study also has inherent limits, such as its retrospective and descriptive design. The relatively small sample size represents a limitation and may limit the generalizability of the findings. Additionally, preoperative ultrasounds are operator-dependent and were performed by different gynecologists, which may have introduced variability and reduced the overall diagnostic reliability of the examinations. Finally, as this is a single-center study, caution should be exercised when extrapolating its results to the general population.

Protective morcellation with containment bags should be considered standard practice during sacrocolpopexy combined with hysterectomy. This study provides reassuring long-term outcomes for patients with incidental endometrial lesions and supports careful postoperative monitoring as a variable alternative to reintervention in selected cases.

## 5 Conclusions

The incidence of occult pathology during sacrocolpopexy associated with supracervical hysterectomy is noteworthy. The rate of occult preinvasive endometrial lesions was 6.2%, and the rate of occult endometrial carcinoma was 0.4%. Several demographic factors, including advanced age, hypertension, postmenopausal status and the use of hormone therapy for breast neoplasia, are associated with an increased risk of occult pathologies. Regardless of preoperative results, morcellation within the free peritoneal cavity should be avoided, any type of morcellation must be performed with protective measures or within a containment bag.

## Data Availability

The raw data supporting the conclusions of this article will be made available by the authors, without undue reservation.
